# Supplementary material for: Photoreduction of Terrigenous Fe‐Humic Substances Leads to Bioavailable Iron in Oceans
Source: Angew Chem Weinheim Bergstr Ger. 2016 Apr 21;128(22):6527–32. doi: 10.1002/ange.201600852 (PMC4949668; doi:10.1002/ange.201600852)
Supplement: Supplementary file 1 — Supplementary [file ANGE-128-6527-s001.pdf]

Angewandte Chemie
Eine Zeitschrift der Gesellschaft Deutscher Chemiker

Supporting Information

Photoreduction of Terrigenous Fe-Humic Substances Leads to Bioavailable Iron in Oceans

*Amir Blazevic, Ewelina Orlowska, Wolfgang Kandioller, Franz Jirsa, Bernhard K. Keppler, Myrvete Tafili-Kryeziu, Wolfgang Linert, Rudolf F. Krachler, Regina Krachler, and Annette Rompel**

ange_201600852_sm_miscellaneous_information.pdf

Photoreduction of Terrigenous Fe–Humic Substances Leads to Bioavailable Fe in Oceans

Amir Blazevic,[a] Ewelina Orlowska,[b] Wolfgang Kandioller,[b] Franz Jirsa,[c] Bernhard K. Keppler,[b] Myrvete Tafili-Kryeziu,[d] Wolfgang Linert,[d] Rudolf F. Krachler,[c] Regina Krachler,[c] Annette Rompel*[a]

[a] Institut für Biophysikalische Chemie, Fakultät für Chemie, Universität Wien, Althanstraße 14, 1090 Wien (Austria)
*corresponding author E-mail: annette.rompel@univie.ac.at; www.bpc.univie.ac.at
[b] Institute of Inorganic Chemistry, Faculty of Chemistry, University of Vienna, Währinger Str. 42, 1090 Vienna (Austria)
[c] Institute of Inorganic Chemistry, Faculty of Chemistry, University of Vienna, Althanstraße 14, 1090 Vienna (Austria)
[d] Institut für Angewandte Synthesechemie, Technische Universität Wien, Getreidemarkt 9/163-AC, 1060 Wien (Austria)

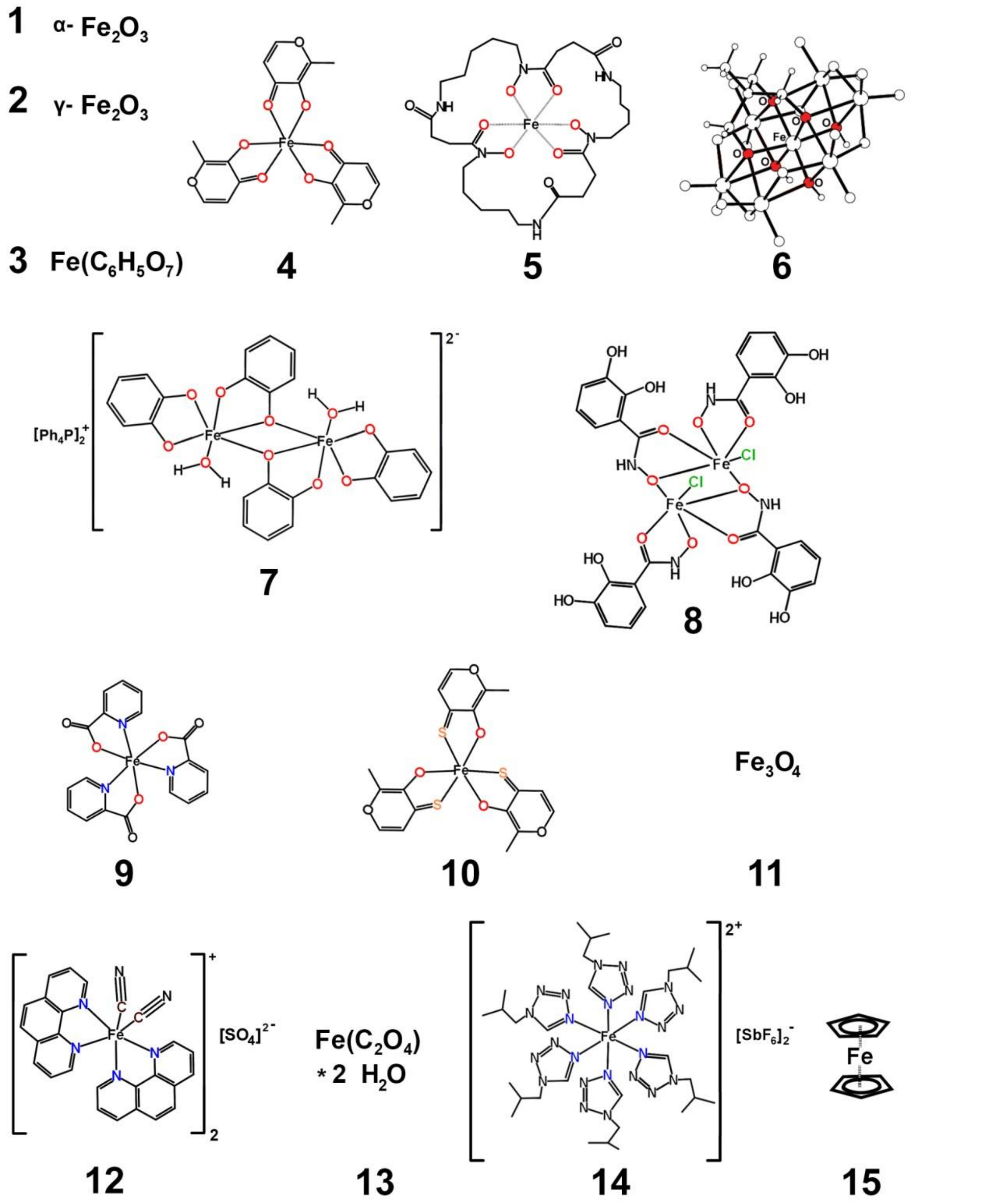

Figure S1. Structures (**1**-**15**) of the Fe model compounds with first coordination shell atoms in color. They are presented in order of the edge energy (high to low) based on the first maximum in the first derivative. Compounds **1**-**10** and **12** contain Fe^{III}, compound **11** contains $Fe^{II/III}$ and compounds **13**-**15** contain Fe^{II}. **1** α-iron(III)oxide hematite (with first coordination shell $Fe^{III}O_6$, Fe_2O_3, Sigma-Aldrich, CAS 1317-60-8), **2** γ-iron(III)oxide maghemite ($Fe^{III}O_6$, Fe_2O_3, Sigma-Aldrich, CAS 1309-37-1), **3** iron(III)citrate ($Fe^{III}O_6$, $C_6H_5FeO_7$, Sigma-Aldrich, CAS 3522-50-7), **4** Fe(maltolato)$_3$ ($Fe^{III}O_6$, $(C_6H_5O_3)_3Fe$) [1], **5** Ferrioxoamine E ($Fe^{III}O_6$, $C_{27}H_{45}FeN_6O_9$, Sigma-Aldrich, CAS 20008-20-2), **6** $Na[C_4H_{12}N]_2[FeMo_6O_{18}(OH)_3\{(OCH_2)_3CNH_3\}7114,17x](OH)\times 6\ H_2O$ ($Fe^{III}O_6$) [2], **7** $(Ph_4P)_2[Fe_2(Cat)_4(H_2O)_2]$ (Cat = catechol) ($Fe^{III}O_6$) [3], **8** Fe_2(hypogallic hydroxamate)$_4Cl_2$ ($Fe^{III}O_5Cl$, $(C_6H_3(OH)_2CHNHO_2)_4Fe_2Cl_2$) [4], **9** Fe(picolinate)$_3$ ($Fe^{III}O_3N_3$, $(C_6H_4NO_2)_3Fe$) [5], **10** Fe(thiomaltolato)$_3$ ($Fe^{III}O_3S_3$, $(C_6H_5O_2S)_3Fe$) [6], **11** Fe_3O_4 magnetite ($Fe^{II/III}O_{4/6,}$ Sigma-Aldrich, CAS 1317-61-9), **12** *cis*-Dicyano-*bis*(1,10-phenanthroline)iron(III) sulfate ($Fe^{III}C_2N_4$, $[Fe(CN)_2(C_{12}H_8N_2)_2]SO_4\times 2\ H_2O$) [7], **13** iron(II)oxalate dihydrate ($Fe^{II}O_6$, FeC_2O_4, Sigma-Aldrich, CAS 6047-25-2), **14** $[Fe^{II}(i4tz)_6][SbF_6]_2$, i4tz = 1-isobutyl-1*H*-tetrazole ($Fe^{II}N_6$) [8] and **15** ferrocene ($Fe^{II}C_6$, $C_{10}H_{10}Fe$) Sigma-Aldrich, CAS 102-54-5).

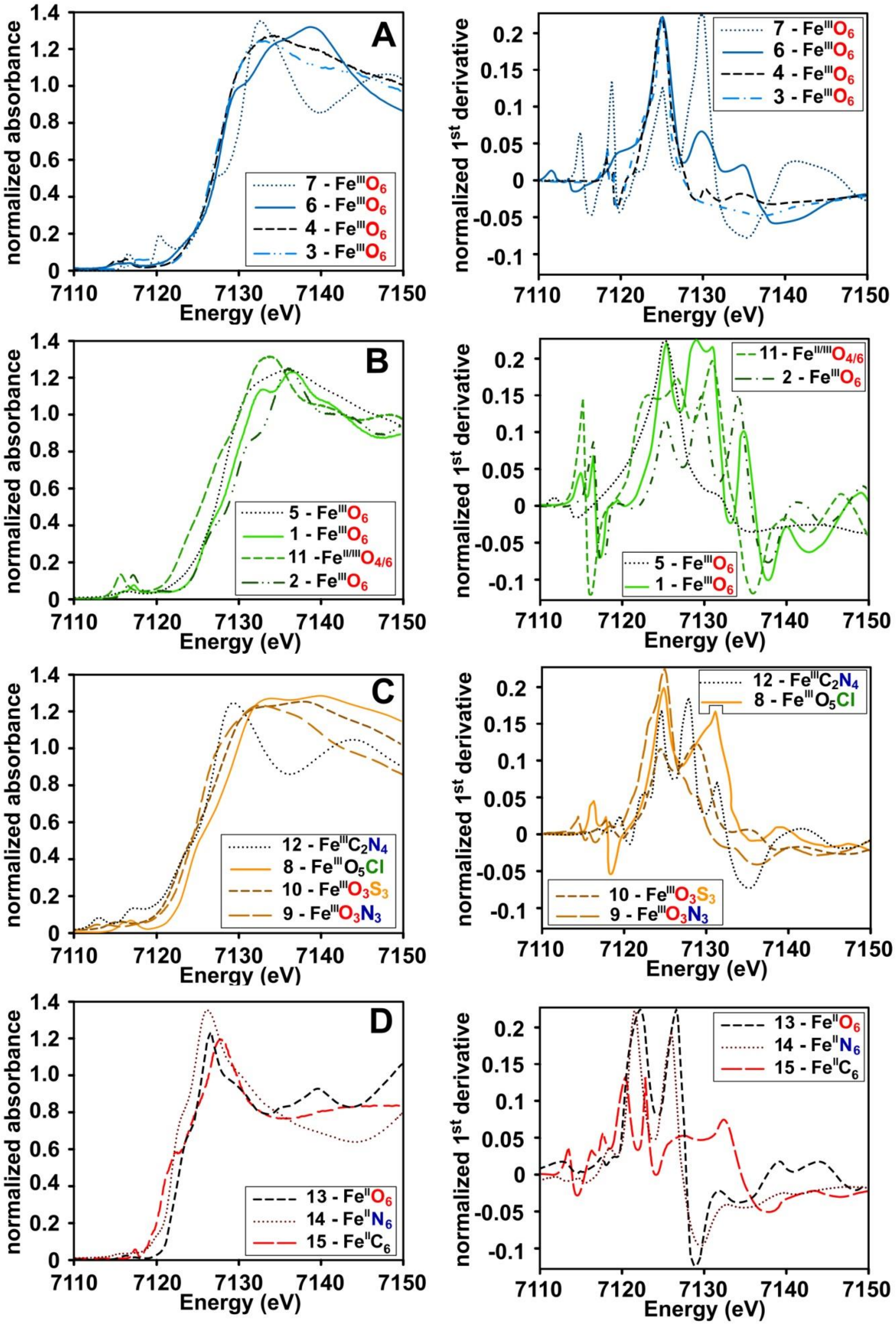

Figure S2. Normalized XANES region of the investigated model compounds and their corresponding first derivatives with $Fe^{III}O_6$ in (A), $Fe^{II/III}O_6$ in (B), mixed Fe^{III} first shells in (C) and Fe^{II} compounds in (D).

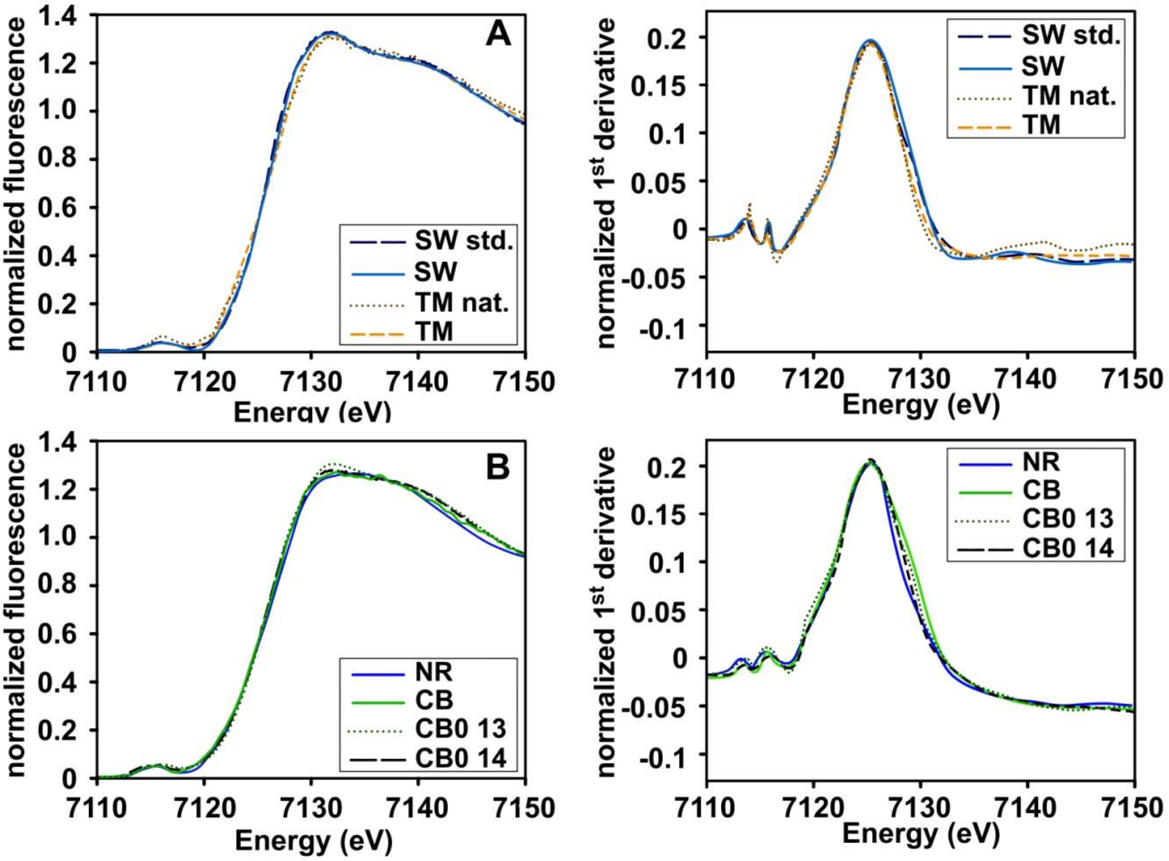

Figure S3. Normalized XANES spectra and first derivatives of the investigated HS samples. TM and SW samples are shown in (A). CB and NR samples are shown in (B).

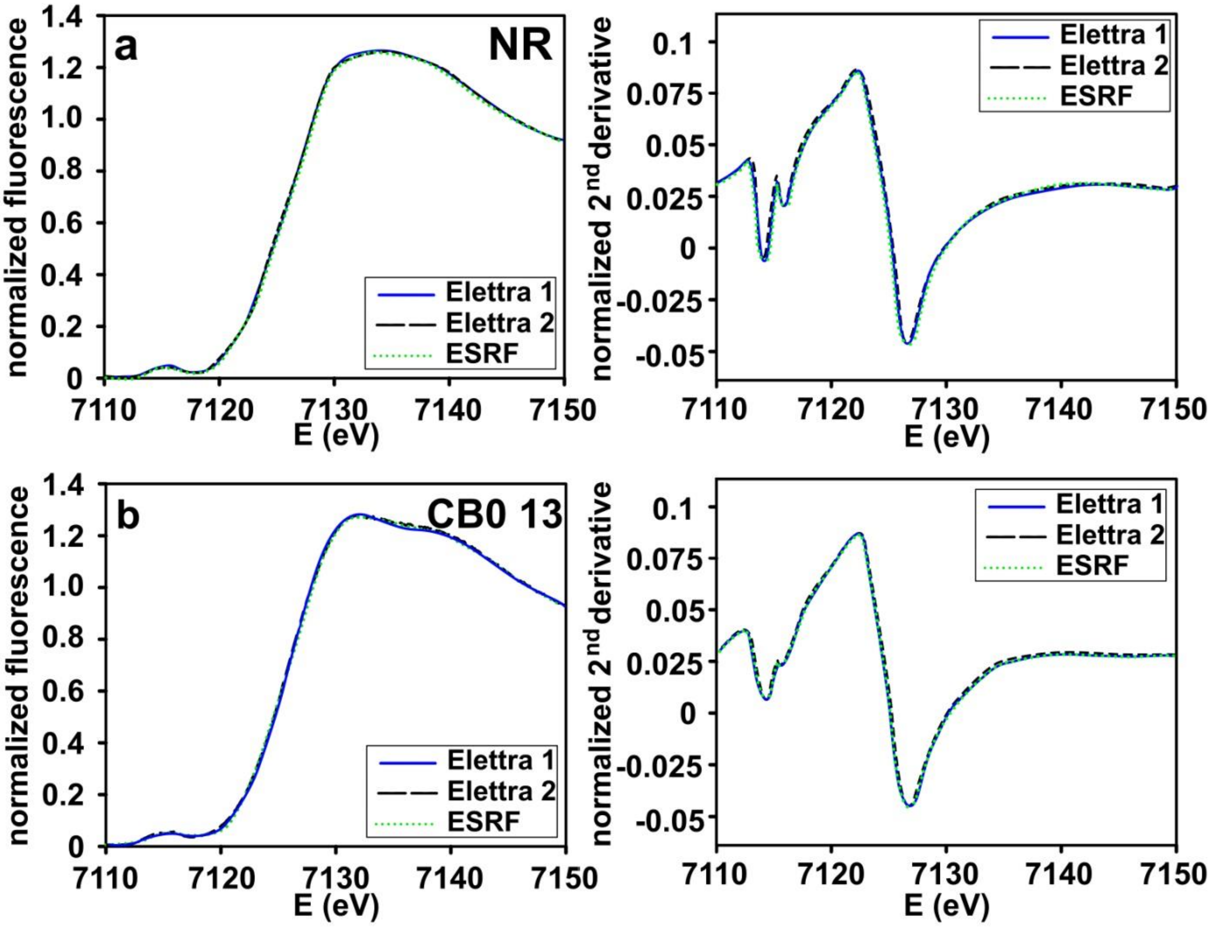

Figure S4. XANES regions and corresponding second derivative of the same NR sample in (a) and CB0 13 sample in (b) showing 2 different scans from Elettra (XAFS beamline)[9] and one scan from the European Synchrotron Radiation Facility (ESRF, beamline ID26)[10] confirming no Fe reduction in any of the samples.

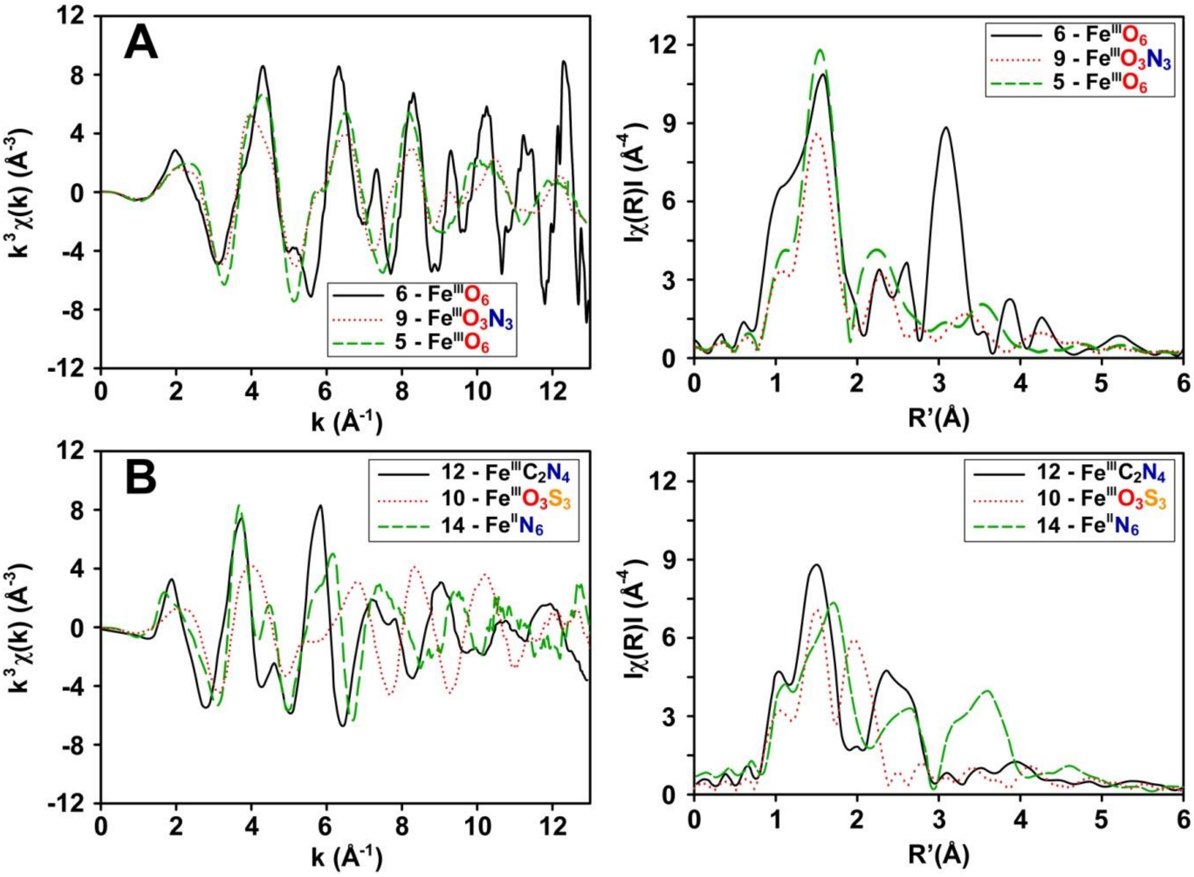

Figure S5. Extracted fine structures and Fourier transforms of model compounds **5**, **6**, **9** in (A) and **10**, **12**, **14** in (B).

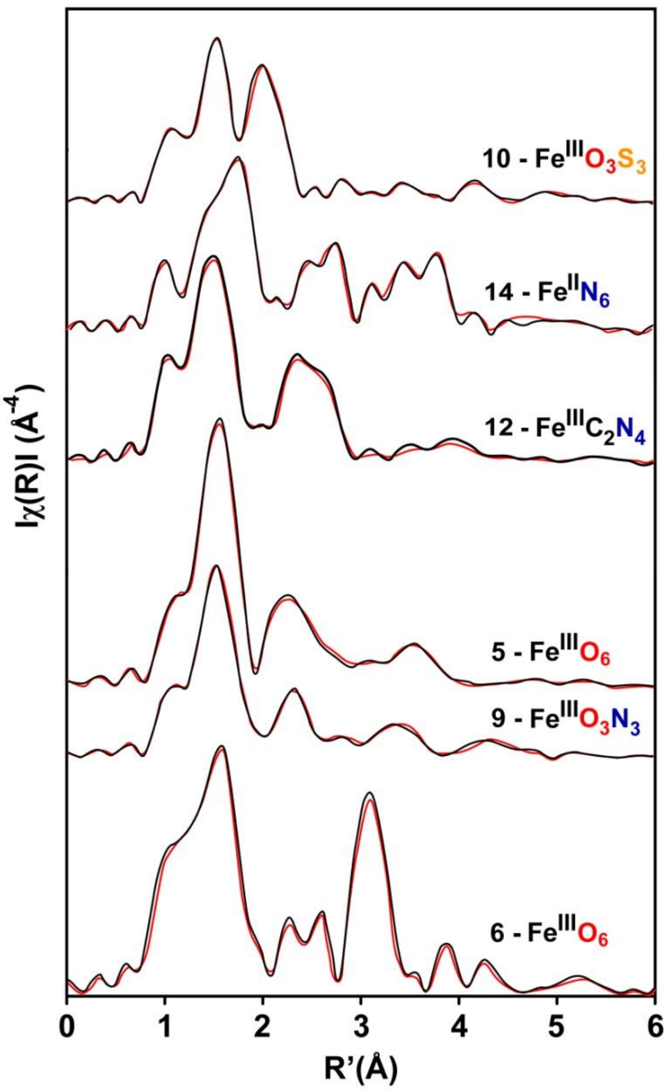

Figure S6. Fourier transform fits of model compounds **5**, **6**, **9**, **10**, **12** and **14**. The solid red line in each sample represents the best fit to crystallographic values.

compound	first coord. shell	geometry	edge energy [eV]	η_{AR}
1	$Fe^{III}O_6$	oct	7125.4	0.06
2	$Fe^{III}O_6$	oct	7125.3	0.06
3	$Fe^{III}O_6$	oct	7125.3	0.06
4	$Fe^{III}O_6$	oct	7125.3	0.06
5	$Fe^{III}O_6$	oct	7125.3	0.06
6	$Fe^{III}O_6$	oct	7125.3	0.06
7	$Fe^{III}O_6$	oct	7125.2	0.06
8	$Fe^{III}O_5Cl$	oct	7124.7	-0.15
9	$Fe^{III}O_3N_3$	oct	7124.1	-0.57
10	$Fe^{III}O_3S_3$	oct	7123.6	-1.11
11	$Fe^{II/III}O_6$	$oct^{II/III}/tet^{III}$	7123.2	-0.46
12	$Fe^{III}C_2N_4$	oct	7123.2	-1.56
13	$Fe^{II}O_6$	oct	7122.1	-0.94
14	$Fe^{II}N_6$	oct	7121.5	-2.20
15	$Fe^{II}C_6$	oct	7120.4	-3.28

Table S1. Edge energies based on the first derivative in the first maximum and the coordination charge for the Fe model compounds.

sample	x-ray irradiation (sec)	natural solar irradiation time on ocean surface* (h)	natural solar irradiation time at 10 m depth * (h)
CB0 13	20	0.4	1.1
	100	1.0	5.3

* The calculations are based on average solar irradiation values given in [11].

Table S2. The average time needed for natural sunlight irradiation to hit an equal size with the same intensity on the ocean surface and at 10 meters depth after 20 and 100 seconds of x-ray exposure. Full reduction of the sample (residence time in the ocean) is expected after the 100 seconds irradiation based on data in Table S3.

sample	R-factor	χ^2	χ^2_{red}	4	5	13
NR	0.009	0.184	0.000348	0.48	0.52	0.00
NR 3/7	0.019	0.548	0.001136	0.47	0.42	0.11
NR 3/21	0.012	0.229	0.000424	0.11	0.25	0.64
NR 5/21	0.011	0.342	0.000624	0.26	0.38	0.36
CB0 13 (10s)	0.012	0.157	0.000308	0.42	0.58	0.00
CB0 13 (20s)	0.023	0.689	0.001374	0.26	0.48	0.26
CB0 13 (30s)	0.017	0.445	0.000964	0.19	0.39	0.42
CB0 13 (40s)	0.016	0.489	0.000910	0.10	0.32	0.58
CB0 13 (50s)	0.018	0.552	0.001098	0.12	0.28	0.60
CB0 13 (60s)	0.012	0.402	0.000790	0.13	0.26	0.61

Table S3. LCF results of model compounds 4, 5 and 13 to the spectra of NR and CB0 13 samples.

compound	first coord. shell	path	R (Å)	R_{cryst}(Å)	Δ R (Å)	σ^2 (Å^2 10^{-3})	E_0 (eV)	fit index (%)
5	$Fe^{III}O_6$	Fe-O	1.99	2.00	0.01	1.42±0.48	2.4±1.8	1.64
6	$Fe^{III}O_6$	Fe-O	1.98	2.00	0.02	1.13±0.51	2.9±2.2	0.44
14	$Fe^{II}N_6$	Fe-N	2.21	2.19	0.02	2.68±1.34	3.5±1.7	2.11
9	$Fe^{III}O_3N_3$	Fe-O	1.95	1.96	0.01	2.55±0.64	3.8±2.1	1.18
		Fe-N	2.15	2.13	0.02			
12	$Fe^{III}C_2N_4$	Fe-C	1.97	1.92	0.05	3.77±1.72	2.1±3.1	4.22
		Fe-N	2.08	1.98	0.10			
10	$Fe^{III}O_3S_3$	Fe-O	1.99	1.97	0.02	3.77±1.76	2.6±2.8	1.31
		Fe-S	2.53	2.50	0.03			

Table S4. First shell fits of the model compounds using theoretical amplitudes and phases provided by the FEFF code [12].

sample	S_o^2 [(a)]	F_i (%)[(b)]	Δ E (eV)[(c)]	path	CN[(d)]	R (Å)[(e)]	σ^2 (Å^2)[(f)]
CB0 13	0.77	1.8	1.4	Fe-O/N (ss)	5.9	2.01	0.0066±0.0004
				Fe-C (ss)	1.8	2.95	0.0054±0.002
				Fe-O/N-C (ms)	3.6[(g)]	3.92	0.0061±0.004
CB0 13 Fe-Fe paths	0.72	11.2	6.8	Fe-O/N (ss)	5.9	2.00	0.0074±0.001
				Fe-C (ss)	2.2	2.91	0.0098±0.004
				Fe ⋯Fe (ss)	1.0	3.38	0.0922±0.050
				Fe-O/N-Fe (ms)	2.2	4.19	0.0738±0.031

[a] amplitude reduction factor [b] F_i is defined as $((\sum(k^3\chi_{exp} - k^3\chi_{fit})^2)/(\sum(k^3\chi_{exp})^2)) \times 100$, $k^3\chi_{exp}$ and $k^3\chi_{fit}$ represents experimental and fitted data points, respectively [c] energy shift parameter [d] coordination number [e] mean bond distance [f] Debye-Waller factor [g] The coordination number of the Fe-C-O/N ms path was correlated 2 × CN of the Fe-C ss path in accordance with e.g. carboxylate structures.

Table S5. Fit results of CB0 13 in R-space with a mononuclear and a polynuclear fitting. ss = single scattering, ms = multiple scattering.

Methods

Field sampling

Humic-rich water samples were collected from the Craggie Burn (CB), a tributary of River Halladale in the Flow Country of Caithness and Sutherland in North Scotland (geographic coordinates: N 58°26′ W 3°54′), and from the Tannermoor brook (TM) in Upper Austria (geographic coordinates: N 48°30′ E 14°52′). Sampling was performed during August. The sampling locations have been described in detail previously [13-16]. Anthropogenic contamination of the sampling sites is regarded as minimal, as the creeks drain unspoiled peatlands and are themselves free-flowing and unpolluted. Water samples were drawn from the surface using a HNO_3 cleaned polyethylene bottle that had been rinsed with ultrapure water. Immediately following collection, the water samples were pumped through 0.2 µm filters (Sartobran 300 Capsules) to remove particulate material, eukaryotes and bacteria, and the filtrates were rapidly filled into acid-cleaned sterile polyethylene bottles to prevent microbial degradation of natural organic Fe chelators in the filtered samples. The samples were stored in the dark at 4°C until further treatment.

Sample preparation

After storage, the suspension was filtered through a syringe filter (Acrodisc 25mm, 0.2µm GHP membrane, PALL). The filtrate was then separated on a Sephadex LH-20 column. Then an ÄKTA purifier was used with conductivity monitoring. A UV detector set to 242 nm was used to detect the natural organic matter (NOM). The eluent was 4.5 % methanol (HPLC grade from Fisher Scientific UK) dissolved in ultrapure water. The sample volume was 2 mL and flow rate was 0.1 mL min^{-1}. The columns used were two series-connected SR 25/100 columns (tube height 1000 mm) from GE Healthcare. After separation, 10 mL fractions were collected using a Frac-900 fraction collector. The NOM fractions were dried at room temperature using a Savant ISS 110 SpeedVac Concentrator from Thermo Scientific. The properties of the CB0 fraction from CB is discussed elsewhere [17]. The sample preparation for CB0 13 and CB0 14 was identical with a one year difference in field sampling. CB0 13/14 and SW std. were measured as solid samples whereas all other samples were re-dissolved in ultrapure water (**Table 1**). SW std. was measured as received from IHSS and TM nat. was measured as collected without any salting out experiments. The pH study conducted on the NR samples was acidified with smallest volume of conc. HCl after the above mentioned preparations to pH 3 or 5. The samples were shaken and left to equilibrate at room temperature for 7 (NR 3/7) and 21 (NR 3/21, NR 5/21) days with exposure to daily natural sunlight light (5 hours per day). **Table 1** shows the HS samples that were measured as wells as their pH, Fe concentration and dissolved organic carbon (DOC) values. DOC and Fe concentration measurements were determined as reported elsewhere [16].

The following model compounds were either purchased from a chemical supply company or synthesized according to the references cited (**Fig. S1**): **1** α-iron(III)oxide hematite (with first coordination shell $Fe^{III}O_6$, Fe_2O_3, Sigma-Aldrich, CAS 1317-60-8), **2** γ-iron(III)oxide maghemite ($Fe^{III}O_6$, Fe_2O_3, Sigma-Aldrich, CAS 1309-37-1), **3** iron(III)citrate ($Fe^{III}O_6$, $C_6H_5FeO_7$, Sigma-Aldrich, CAS 3522-50-7), **4** Fe(maltolato)$_3$ ($Fe^{III}O_6$, $(C_6H_5O_3)_3Fe$) [1], **5** Ferrioxoamine E ($Fe^{III}O_6$, $C_{27}H_{45}FeN_6O_9$, Sigma-Aldrich, CAS 20008-20-2), **6** $Na[C_4H_{12}N]_2[FeMo_6O_{18}(OH)_3\{(OCH_2)_3CNH_3\}x](OH)\times 6\ H_2O$ ($Fe^{III}O_6$) [2], **7** $(Ph_4P)_2[Fe_2(Cat)_4(H_2O)_2]$ (Cat = catechol) ($Fe^{III}O_6$) [3], **8** Fe_2(hypogallic hydroxamate)$_4Cl_2$

($Fe^{III}O_5Cl$, $(C_6H_3(OH)_2CHNHO_2)_4Fe_2Cl_2$) [4], **9** Fe(picolinate)$_3$ ($Fe^{III}O_3N_3$, $(C_6H_4NO_2)_3Fe$) [5], **10** Fe(thiomaltolato)$_3$ ($Fe^{III}O_3S_3$, $(C_6H_5O_2S)_3Fe$) [6], **11** Fe_3O_4 magnetite ($Fe^{II/III}O_{4/6}$, Sigma-Aldrich, CAS 1317-61-9), **12** *cis*-Dicyano-*bis*(1,10-phenanthroline)iron(III) sulfate ($Fe^{III}C_2N_4$, $[Fe(CN)_2(C_{12}H_8N_2)_2]SO_4 \times 2\ H_2O$) [7], **13** iron(II)oxalate dihydrate ($Fe^{II}O_6$, FeC_2O_4, Sigma-Aldrich, CAS 6047-25-2), **14** $[Fe^{II}(i4tz)_6][SbF_6]_2$, i4tz = 1-isobutyl-1*H*-tetrazole ($Fe^{II}N_6$) [8] and **15** ferrocene ($Fe^{II}C_6$, $C_{10}H_{10}Fe$Sigma-Aldrich, CAS 102-54-5). The model compounds were diluted in BN (Sigma Aldrich, CAS 10043-11-5, 99.5%) with a highly uniform optical thickness, placed into PEEK sample holders, and sealed with Kapton foil. All samples were flash frozen with liquid nitrogen. The BN preparations were prepared for a calculated absorption of about 1 absorbance unit according to standard methods [18]. The liquid and solid HS samples were placed in Kapton foil sealed PEEK sample holders and flash frozen with liquid nitrogen. The prepared HS and model compounds were used for both XAS and VtC-XES experiments.

Data Collection

ESRF

XANES was measured in high-energy-resolution fluorescence-detection (HERFD) mode using a double-crystal monochromator equipped with Si(111) and Si(311) crystals at beamline ID26. The electron energy was 6.0 GeV and the ring current varied between 150 and 200 mA. Higher harmonics were rejected by two Si mirrors. An avalanche photodiode was used as the X-ray photon detector. XANES spectra were acquired by scanning the monochromator in continuous mode with a step size of 0.05 eV. The samples were scanned from 7100 to 7200 eV. For the valence-to-core XES measurements, samples were oriented at 45° with respect to the incident beam. The energy was tuned just above the Fe K-edge (7.2 keV) and the maximum incident flux was 10^{13} photons/s. An avalanche photodiode was also used as the X-ray photon detector. The beam size on the sample was 0.4 mm horizontal and 1 mm vertical. Two Si mirrors operating in total reflection rejected higher harmonics. The energy bandwidth of the x-ray emission detection was 0.6 eV. The X-ray emission was measured by means of a spherically bent (R = 1000 mm) Si wafer with (440) orientation arranged in a Rowland geometry. The scattering plane is horizontal with the analyzer crystal positioned at a 90° scattering angle, i.e., along the polarization vector of the linear polarized incident X-ray beam. Three ion chambers were applied which measured the incident, transmitted, and reference beam intensities, respectively. Fe foil (5 µm) was applied for energy scale calibration which was placed between the second and third ion chambers so that the absorption spectrum of the foil was recorded simultaneously. The energy value of the first maximum in the first derivative for Fe was taken as 7112 eV. All experiments were conducted at cryogenic temperatures at 90 K.

Elettra

XANES and EXAFS measurements of model compounds and HS samples were performed at the XAFS beamline. The model compounds were measured in transmission mode (90° orientation with respect to the incident beam), and the HS samples were collected in fluorescence mode (45° orientation with respect to the incident beam). XANES and EXAFS spectra were recorded using a Si(111) monochromator. The samples were scanned from 6800 to 7660 eV with a step size of 0.1 eV. The electron energy was 2.0 GeV and the ring current was 309 mA. Higher harmonics were rejected by two Si mirrors.

Samples were measured in transmission mode by using an ionization chamber filled with a mixture of Ar, N_2, and He and total electron yield detector. For measurements in fluorescence mode, a silicon drift detector was used. Three ion chambers were applied which measured the incident, transmitted, and reference beam intensities, respectively. Fe foil (5 µm) was applied for energy scale calibration which was placed between the second and third ion chambers so that the absorption spectrum of the foil was recorded simultaneously. The energy value of the first maximum in the first derivative for Fe was taken as 7112 eV. All experiments were conducted at cryogenic temperatures at 90 K.

Data analysis

The program packages ATHENA [19], ARTEMIS [19], IFEFFIT [20], FEFF [12, 21], PySpline [22], Sixpack [23], and PyMCA [24] were applied for the XAS data analysis. Initially careful radiation damage studies were performed by studying spectra collected at different time intervals. Only spectra that showed no signs of decomposition were further processed.

XANES analysis

The pre-edge background was removed by a linear approximation in the range of −20 eV to −130 eV before the edge. This baseline was subtracted from the entire spectrum. The normalization of the Fe model compounds and humic substance samples was accomplished by fitting a quadratic polynomial in the post edge region from 120 eV to 550 eV and 60 eV to 240 eV post the edge, respectively. The fitted functions were extrapolated to the first absorption maximum and the absorbance was set to unity there. The edge position was determined as the maximum in the first derivative of the spectrum (inflection point of the steeply rising edge).

The XANES spectra were analyzed (Athena and Sixpack) using principal component analysis (PCA) and linear combination fitting (LCF) as described elsewhere [25]. PCA was first performed to define the number of significant orthogonal components within the HS sample dataset followed by target transformations to determine the most likely species present in the samples. This was based on the SPOIL value and the *F* test [26].

EXAFS analysis

EXAFS analysis followed standard procedures described elsewhere [18, 20]. The ab initio amplitude and phases were provided by the program package FEFF8 [12]. The crystallographic values were used as an input for an initial structural model. The EXFAS signals were extracted using ATHENA and PySpline. ARTEMIS and IFEFFIT were used to fit the FEFF8 amplitudes and phases. The fitting procedure of the model compounds was performed on the Fourier Transform real part between 1 and 6 Å using a Hanning window (dk-value = 1). It was optimized for *k* -weights 1, 2, and 3. The model compounds were fitted using all scattering paths as calculated by Atoms. In order to not exceed the number of fitting parameters, the identity and number of backscatterers were fixed to the crystallographic values. The known distances were taken as a starting point for the fitting analysis. Four separate mean-square displacement factors were used in total; one for the six first-shell Fe-X paths, two for the second shell paths (separating light and heavy elements) and one for all remaining paths. A single ΔE parameter was used for all the paths in a given sample, but it was allowed to vary between samples. The interatomic distance parameter, *R*,

was allowed to vary for all paths. The results of the first shell fits of the model compounds using theoretical amplitudes and phases are presented in Table S4 and graphically as non-phase shifted FT in Figure S6. The distances are given as the average fitted distances for each atom type. All compounds are in good agreement with crystallographic values, thus, the extracted amplitude and phase shift is justified.

The fitting procedure of CB0 13 was performed on the Fourier Transform real part between 1 and 6 Å using a Hanning window (dk-value = 1). It was optimized for k -weights 1, 2, and 3. The coordination number was allowed to vary for Fe–O/N single scattering paths in the first coordination shell together with the mean-square displacement factor. Multiple scattering of the oxygen atoms in the first shell was fit in the same way as previously reported for Fe^{III} bound to fulvic acid [27]. For second shell paths, only paths that decreased the χ^2 value significantly (> 1.5) was included while the fitted Debye–Waller factors remained in a reasonable range (< 0.01 Å^2) [18]. Thus, no Fe-Fe paths could be included. Compound **4** (octahedral, $Fe^{III}O_6$) was used for fitting of first shells and compounds **1** (octahedral, $Fe^{III}O_6$), **5** (octahedral, $Fe^{III}O_6$) and **9** (octahedral, $Fe^{III}O_3N_3$) for fitting of second and third shell paths. The number of independent points in all fits is according to the Nyquist theorem [28] in the range 14-17. This clearly exceeds the maximum number of free variables in our fitting procedure, which was 11 as most making sure that the EXAFS spectrum is not overfitted.

VtC-XES analysis

Because of the strong $K\beta_{1,3}$ line at lower emission energies, the valence-to-core transitions are superimposed on a background. By fitting Voigt line profiles to the $K\beta_{1,3}$ line, the background was removed. The VtC-XES signal is weaker by a factor 2−3 compared to the background which may lead to a small error in the Kβ′′ region during background subtraction.

Coordination charge calculation

The exact position of the edge in the XANES is dependent on several features like valence, electronegativity of the first shell atoms, their coordination number and the formal oxidation state of the central atom. To take these factors into consideration, Batsanov [29] introduced the concept of the coordination charge η, which can be calculated according to the following formula:

$$\eta = m - \sum_k n_k\, c_k \qquad (1)$$

where m is the formal oxidation state of the central metal, c_k is the degree of covalence of a bond k and n_k is the number of such bonds [30]. The edge position is shifted to a higher energy when the electronegativity of the neighboring atoms is increased [31]. The degree of covalence c_k is defined as $1 - I_k$, where I_k is the ionicity of that bond. The ionicity is calculated using Pauling's formula (eq. 2):

$$I_k = 1 - \exp\left[-\frac{1}{4}\left(\chi_M - \chi_L\right)^2\right] \qquad (2)$$

The iconicity depends on the electronegativity of the central Fe atom χ_M and the electronegativity of the ligand atom χ_L [31]. The coordination charge is calculated as in eq. 1 applying I_k calculated as in eq. 2. The change in electronegativity due to changes in

hybridization state was not considered in these calculations. The calculations give the following ionicities and covalencies:

Table S6. Calculated ionicities and covalencies for the different ligands available in the Fe model compounds.

element	Z	ionicity	covalency
carbon	6	0.12	0.88
oxygen	8	0.51	0.49
nitrogen	7	0.30	0.70
sulfur	16	0.12	0.88
chlorine	17	0.30	0.70

Full citation details of abbreviated referecences in the main manuscript

R. Krachler, R. F. Krachler, G. Wallner, S. Hann, M. Laux, M. F. Cervantes Recalde, F. Jirsa, E. Neubauer, F. von der Kammer, T. Hofmann, B. K. Keppler, *Mar. Chem.* 2015, *174*, 85-93.

P.-S. Kuhn, L. Cremer, A. Gavriluta, K. K. Jovanović, L. Filipović, A. A. Hummer, G. E. Büchel, B. P. Dojčinović, S. M. Meier, A. Rompel, S. Radulović, J. B. Tommasino, D. Luneau, V. B. Arion, *Chem. Eur. J.* 2015, *21*, 13703-13713.

E. G. Anassontzis, A. E. Ball, A. Belias, A. Fotiou, G. Grammatikakis, H. Kontogiannis, P. Koske, S. Koutsoukos, V. Lykoussis, E. Markopoulos, A. Psallidas, L. K. Resvanis, I. Siotis, S. Stavrakakis, G. Stavropoulos, V. A. Zhukov, *Astropart. Phys.* 2010, *34*, 187-197.

References

[1] M.T. Ahmet, C.S. Frampton, J. Silver, Journal of the Chemical Society, Dalton Transactions, (1988) 1159-1163.
[2] A. Blazevic, E. Al-Sayed, A. Roller, G. Giester, A. Rompel, Chemistry – A European Journal, 21 (2015) 4762-4771.
[3] V.A. Grillo, G.R. Hanson, D. Wang, T.W. Hambley, L.R. Gahan, K.S. Murray, B. Moubaraki, C.J. Hawkins, Inorganic Chemistry, 35 (1996) 3568-3576.
[4] E. Orlowska, W. Kandioller, H. Wiesinger, R. Krachler, F. Jirsa, B.K. Keppler, ARC Advances.
[5] S. Kiani, A. Tapper, R.J. Staples, P. Stavropoulos, Journal of the American Chemical Society, 122 (2000) 7503-7517.
[6] J.A. Lewis, D.T. Puerta, S.M. Cohen, Inorganic Chemistry, 42 (2003) 7455-7459.
[7] F. Werner, G. Kickelbick, W. Linert, Journal of Coordination Chemistry, 54 (2001) 167-174.
[8] M. Tafili-Kryeziu, M. Weil, T. Muranaka, A. Bousseksou, M. Hasegawa, A. Jun, W. Linert, Dalton Trans., 42 (2013) 15796-15804.
[9] C. Andrea Di, A. Giuliana, M. Marco, P. Emiliano, N. Nicola, C. Andrea, O. Luca, Journal of Physics: Conference Series, 190 (2009) 012043.
[10] C. Gauthier, V.A. Sole, R. Signorato, J. Goulon, E. Moguiline, Journal of Synchrotron Radiation, 6 (1999) 164-166.
[11] W. Larcher, Ökophysiologie der Pflanzen, Verlag Eugen Ulmer, Stuttgart (Hohenheim), 2001.
[12] A.L. Ankudinov, B. Ravel, J.J. Rehr, S.D. Conradson, Physical Review B, 58 (1998) 7565-7576.

[13] R. Krachler, F. von der Kammer, F. Jirsa, A. Süphandag, R.F. Krachler, C. Plessl, M. Vogt, B.K. Keppler, T. Hofmann, Global Biogeochemical Cycles, 26 (2012) GB3024.
[14] R. Krachler, F. Jirsa, S. Ayromlou, Biogeosciences, 2 (2005) 311-315.
[15] R. Krachler, R.F. Krachler, F. von der Kammer, A. Süphandag, F. Jirsa, S. Ayromlou, T. Hofmann, B.K. Keppler, Science of The Total Environment, 408 (2010) 2402-2408.
[16] F. Jirsa, E. Neubauer, R. Kittinger, T. Hofmann, R. Krachler, F. von der Kammer, B.K. Keppler, Limnologica - Ecology and Management of Inland Waters, 43 (2013) 239-244.
[17] R. Krachler, R.F. Krachler, G. Wallner, S. Hann, M. Laux, M.F. Cervantes Recalde, F. Jirsa, E. Neubauer, F. von der Kammer, T. Hofmann, B.K. Keppler, Marine Chemistry, 174 (2015) 85-93.
[18] S.D. Kelly, D. Hesterberg, B. Ravel, Analysis of Soils and Minerals Using X-ray Absorption Spectroscopy, in: Methods of Soil Analysis. Part 5. Mineralogical Methods, American Society of Agronomy, Madison, WI, 2008, pp. 387-463.
[19] B. Ravel, M. Newville, Journal of Synchrotron Radiation, 12 (2005) 537-541.
[20] M. Newville, Journal of Synchrotron Radiation, 8 (2001) 322-324.
[21] J.J. Rehr, R.C. Albers, Reviews of Modern Physics, 72 (2000) 621-654.
[22] A. Tenderholt, B. Hedman, K.O. Hodgson, PySpline: A modern, cross-platform program for the processing of raw averaged XAS edge and EXAFS data, in: B. Hedman, P. Painetta (Eds.) X-Ray Absorption Fine Structure-XAFS13, Amer Inst Physics, Melville, 2007, pp. 105-107.
[23] S.M. Webb, Physica Scripta, 2005 (2005) 1011.
[24] V.A. Solé, E. Papillon, M. Cotte, P. Walter, J. Susini, Spectrochimica Acta Part B: Atomic Spectroscopy, 62 (2007) 63-68.
[25] S. Beauchemin, D. Hesterberg, M. Beauchemin, Soil Science Society of America Journal, 66 (2002) 83-91.
[26] E.R. Malinowski, Analytica Chimica Acta, 103 (1978) 339-354.
[27] J.W.J. van Schaik, I. Persson, D.B. Kleja, J.P. Gustafsson, Environmental Science & Technology, 42 (2008) 2367-2373.
[28] L. Brillouin, Science and Information Theory, Dover Publications, 2004.
[29] S.S. Batsanov, Electronegativity of Elements and Chemical Bonds, Nauka, Novosibrsk, Russia 1962.
[30] S.P. Cramer, T.K. Eccles, F.W. Kutzler, K.O. Hodgson, L.E. Mortenson, Journal of the American Chemical Society, 98 (1976) 1287-1288.
[31] J. Wong, F.W. Lytle, R.P. Messmer, D.H. Maylotte, Physical Review B, 30 (1984) 5596-5610.
